# British Home for Incurables, Clapham Rise

**Published:** 1892-11-26

**Authors:** 


### HOSPITAL MEETINGS.

### BRITISH HOME FOR INCURABLES, CLAPHAM RISE.

Those who fancy that a home for incurable cases is necessarily a dismal kind of an institution had better visit the one at Clapham without delay, as it will rapidly disabuse their minds of this impression.

Brightness, cleanliness, and order are the prevailing characteristics of the "British Home." The brilliantly polished grates contain cheerful fires, which even on foggy days give a pleasant look to rooms, peopled by helpless invalids. There are so many sitting-rooms, all facing the thoroughfare, from which a small enclosed plot of land separates them. Clapham Road is an interesting one, for there is always motion in it of vehicles or foot passengers, and movement, from force of contrast, is immensely attractive to those spectators whom fate has debarred from all active pursuits.

Nearly all the patients at the British Home are quite incapable of helping themselves; they are washed and dressed, and then they are transferred to the cheery sitting-rooms, where they remain on couches or in chairs until the hour comes for their return to bed. The more active can just manage to wheel themselves about in their easily-moved wheel-chairs, but to many even this amount of capacity is denied. Therefore the tender care needful can hardly be over-estimated for it is incessant, and the patience and gentleness demanded from the attendants are unlimited.

When we see the inmates cheerful, warm, and neat, we can draw satisfactory conclusions as to the ability of the staff. For example, neatness is not always attained at home for these helpless persons, they frequently have a forlorn appearance when it is no longer in their own power to keep themselves orderly. Here, however, the careful adjustment of cap or shawl, of rug or quilt, gives the visitor a pleasant and instinctive conception of the fitness of each article of attire. The patients look thoroughly well-dressed and comfortably placed, each has her own special trifles within reach, and everybody is situated so as to share the advantages of window and fire. There are no poor creatures in dark, dull corners, they are all in the full enjoyment of such daylight as the season permits. Most of the bed-rooms have bow-windows, giving a good view of the extensive gardens, which are a valuable possession in summer days.

The men are looked after by male attendants, the senior of whom has been employed by the institution for twelve years. The system appears to work well, and these attendants also do the heavy work of lifting and carrying the female patients with great success, and definite advantage to the nurses.

The election of new patients on the 10th inst. excited much attention beforehand amongst the inmates, and when we visited the home we found them discussing the subject with lively interest, and especially hoping for fine weather for that occasion.

Certainly their wishes were granted, if an absence of rain can be taken to constitute a fine day, but the fog on Thursday, November 10th, was somewhat disconcerting to travellers in the metropolis.

The half-yearly general meeting took place at the Cannon Street Hotel at noon, and it was followed by the election of eleven patients from the combined list of in and out-patient candidates. The chairman, Francis A. Bevan, Esq., explained that ten of these would be chosen by election, and one would be the candidate whose name had been longest on the list.

Mr. Bevan then spoke of the event which might be accounted as the most important of the past year, namely, the laying of the foundation-stone of the new buildings at Streatham. They had a clear sum of £3,000 already in hand towards the expenses of erecting the new home, but they wanted £7,000 to complete the work thoroughly.

The Princess of Wales had handed in a further sum of £150 to the secretary, Mr. Gofton Salmond, part of the proceeds of the sale of Canon Fleming's sermon on the occasion of the death of the Duke of Clarence.

Special attention was called by the Chairman to the class of people from whom the candidates came, and their particular claims. He said they were all the children of hard-working, middle class parents, many of them farmers and others who had been reduced to poverty through no fault of their own.

There was no financial report offered as that is given at the annual meeting in May. Notice was given of a sale of the patients' work, for their own benefit, to be held on December 7th and 8th, at the Institution, Clapham Road. The Lady Sudeley has kindly consented to open the sale at half-past two on the first day.

A concert was also announced to take place in December for the benefit of the British Home for Incurables.

